# The Effects of Sample Transport by Pneumatic Tube System on Routine Hematology and Coagulation Tests

**DOI:** 10.1155/2018/6940152

**Published:** 2018-07-02

**Authors:** Devi Subbarayan, Chidambharam Choccalingam, Chittode Kodumudi Anantha Lakshmi

**Affiliations:** Department of Pathology, Chettinad Health City and Research Institute, Kelambakkam, Chennai, Tamil Nadu, India

## Abstract

**Background:**

Automation helps improve laboratory operational efficiency and reduce the turnaround time. Pneumatic tube systems (PTS) automate specimen transport between the lab and other areas of the hospital. Its effect on complete blood count (CBC) and coagulation is still controversial.

**Aim:**

To study the effects of pneumatic tube system sample transport on complete blood count and coagulation parameters to compare them with hand delivered samples.

**Methods:**

75 paired samples for complete blood count and 25 paired samples for coagulation analysis were compared between samples sent via pneumatic tube system and hand delivered system.

**Results:**

PTS showed significant decrease in red cell indices such as MCV and RDW and increase in MCHC. Other red cell parameters and WBC parameters showed no statistical significant difference. Statistically significant increase in platelet count was observed with PTS samples. However, these differences were clinically insignificant. No significant effect of PTS was found in PT and APTT samples compared to the hand delivered samples.

**Conclusion:**

Despite statistically significant changes in RBC parameters such as MCV, RDW, and MCHC and platelet count, these changes were clinically insignificant. Hence, blood samples for CBC and coagulation assay can safely be transported via our hospital's PTS. However, further studies on platelet count are warranted to ensure safe transport and accuracy of the results.

## 1. Introduction

To ensure the fastest possible turnaround time in laboratory analysis, the specimens should be delivered to the clinical laboratory quickly and safely. One such system used for sample transport is pneumatic tube system (PTS).

PTS automate specimen transport by vacuum and pressure between the lab and other areas of the hospital. During transport, the sample integrity can be affected by acceleration, deceleration forces, and radial gravity forces. Steige and Jones have stated that each pneumatic tube system must be individually evaluated because of the differences between each of pneumatic tube systems [1].

Previous studies have shown the changes in platelet aggregation and biochemical parameters such as elevated lactate dehydrogenase, alterations in serum potassium, serum haemoglobin, and arterial blood gas analysis due to PTS transport [2–6]. Few studies have shown shortening of activated partial thromboplastin time (APTT) and changes in mean platelet component (MPC) [3, 7]. Although the effect of PTS on biochemical changes and hemolysis has been studied widely, its effect on complete blood count (CBC) and coagulation samples is still controversial. Hence, we undertook this study to evaluate the effects of PTS on complete blood count and coagulation.

## 2. Aims And Objectives

This paper aims to study the effects of pneumatic tube system sample transport on complete blood count and coagulation parameters.

## 3. Materials And Methods

The study was carried out after obtaining ethical clearance from institutional ethical committee and written informed consent from the study subjects.

75 randomly selected paired samples for CBC and 25 random paired samples for coagulation assay were collected during 2-month period from June 2017 to July 2017.

### 3.1. Sample Collection

Specimens were collected from outpatient collection center, ICUs, and wards. Specimens were collected by standard venipuncture under septic precautions.

75 duplicate venous samples of 3 ml blood were obtained in tripotassium ethylenediaminetetraacetic acid (K3-EDTA) vacutainer (Greiner Bio-One vacutainer). 25 duplicate samples of 2.7 ml blood were collected in 3.2% sodium citrate vacutainer (Becton Dickinson vacutainer) for coagulation. Collected samples were separated into two groups.

### 3.2. Sample Transport

Group 1 samples were immediately transported to the laboratory through PTS. The PTS used in this study was Swisslog's PTS (Swisslog Rohrpostsysteme GmbH, Hansacker 5-7, Westerstede, Germany). The system works electronically with TranspoNet software process to maximize its efficiency. Two types of carriers were used in this system: one for transportation of the sample which is leak proof provided with a special foam tube carriers and the other for sending request forms. The samples from various stations (ground floor, first floor, second floor, third floor, fourth floor, and fifth floor) to central collection laboratory are programmed at a speed of 5 m/s to reach the laboratory within 50 seconds, 53 seconds, 54 seconds, 56 seconds, 57 seconds, and 59 seconds, respectively.

Group 2 samples were hand delivered to the laboratory by personnel immediately.

CBC values were obtained from LH 780 automated analyzer (Beckman Coulter, India) using electrical impedance for total leukocyte count (TC), red blood cells count (RBC), and platelet count (PLT) and VCS (volume, conductance, and light scatter) technology for differential leukocyte count.

For coagulation studies, samples were immediately centrifuged at 2000 g at 15 mins at room temperature. Platelet-poor plasma was obtained. The PT and APTT assays were done on semiautomated photo optical coagulation analyzer (Sysmex CA-50) using reagents Thromborel S and Actin FSL, respectively.

CBC values and coagulation assays for PTS and hand delivered samples were entered in the data sheet.

### 3.3. Statistical Analysis

SPSS version 17.00 was used for statistical analysis. Mean, mean difference, standard deviation, and standard error of mean were calculated. All parameters between two groups were compared using paired* t-*test for statistical significance.* P* value < 0.05 was considered as statistically significant.

## 4. Results

75 paired samples for CBC were compared for PTS and hand delivered systems. The RBC parameters such as red blood corpuscle (RBC) count, hemoglobin (Hb), and mean cell hemoglobin (MCH) were comparable between two transport systems. However, there was a statistically significant decrease in MCV (mean corpuscular volume) and RDW (red cell distribution width) and increase in MCHC (mean corpuscular hemoglobin concentration) in PTS samples as compared to hand delivered samples (Table 1).

The estimated WBC parameters such as total count and differential count were similar between the two transport systems with no statistically significant difference.

Statistically significant elevation of platelet count was noted in PTS samples with mean difference of 0.135x10^9^/l with 95% confidence interval of 0.073–0.197. MPV showed statistically significant difference between the PTS and hand delivered system, wherein the mean difference was 0.18 with 95% confidence interval (0.32908–0.03359) (Table 1).

25 paired PTS and hand delivered samples were analyzed for PT and APTT. No statistically significant results were found for PT and APTT values between the two transport systems used (Table 2).

## 5. Discussion

PTS are widely used in hospitals to transport blood specimens to the clinical laboratory for most biochemical and hematological analyses. The present study aims to know the effects of transport of blood samples sent through Swisslog's PTS compared with hand delivered samples on complete blood count and coagulation using Beckman Coulter LH780 automated analyzer and Sysmex CA 50 semiautomated coagulation analyzer, respectively.

In the present study, the estimated RBC parameters such as RBC count, Hb, and MCH and WBC parameters such as total count and differential count were comparable between two transport systems (PTS and hand delivered samples). However, red cell indices such as MCV and RDW showed statistically significant decrease in PTS samples, while MCHC showed significant increase in PTS samples. Though there is significant statistical difference in these parameters, the present study observed mean difference percentage of 0.9%, 1%, and 2.1% for MCV, MCHC, and RDW, respectively, which is well below the clinically significant difference of 4-5% [8].

In this study, samples sent via PTS gave a statistically significant increase in platelet count which can be attributed to the fact that abrupt changes in force during transport can cause fragmentation of platelets. The present study observed increase in platelet count with significant decrease in MPV which had inverse relationship. However, though the present study observed statistically significant increase in platelet count, the mean difference (0.135 = 4.9%) is clinically insignificant. To be considered clinically significant, the difference should be 10–15% [8], which was not so in the present study.

Previous studies on CBC did not find any significant difference between the two transport systems [3, 7, 9–11]. Lee et al.'s study [12] demonstrated statistically significantly low MPV values with PTS samples compared to hand delivered samples. However, their study did not observe any significant difference in platelet count. Kratz et al. [7] showed statistically significant but clinically insignificant difference in MPC. So, this might turn out to be the first study that shows statistically significant effect of PTS on platelet count over hand delivered samples which may indicate the need for further studies.

The PT and APTT values were comparable between the two transport systems. Weaver et al. [3] observed statistically significant shortening of mean partial thromboplastin time (PTT) in samples sent through PTS. However, it was found to be clinically insignificant, since the difference was within the standard deviation of the method used. They did not find any significant difference for PT between these two systems. Kratz et al. [7] studied the effects of PTS on PT, PTT, and fibrinogen, and fibrin monomers showed no statistically significant difference.

There are few limitations in our study. The present study did not compare the effects of PTS at different levels of distance and also lack of significant number of samples with abnormal values to evaluate the effects of PTS in these cases, especially with abnormal platelet counts.

## 6. Conclusion

Based on the results, PTS showed significant decrease in red cell indices such as MCV and RDW and increase in MCHC. Samples sent via PTS gave statistically significant increase in platelet count. However, these differences were clinically insignificant. No significant effect of PTS was found in PT and APTT samples compared to the hand delivered samples. Hence, absence of clinically significant changes with samples sent via PTS in the present study concludes that blood samples for CBC and coagulation assay can safely be transported via our hospital's PTS.

Further studies on platelet counts and using different levels of distance should be done to ensure safe transport and accuracy of the results.

## Figures and Tables

**Table 1:** Summary of the differences in CBC between pneumatic tube samples and hand delivered samples.

S. no.	Paired samples	Mean difference	Standard deviation of mean difference	95% confidence interval of mean difference		*P* value
				Lower	Upper	
1	RBC P-RBC M (x10^12^/l)	-.03	.21	-.08	.01	.11
2	HB P-HB M (g/dl)	-.01	.24	-.06	.05	.84
3	MCV P-MCV M (fl)	-.82	1.9	-1.28	-.37	.001
4	MCH P-MCH M (pg)	.03	.41	-.06	.12	.500
5	MCHCP-MCHC M (g/dl)	.36	.84	.17	.56	<0.001
6	RDW P-RDW M (%)	-.33	.67	-.49	-.18	<0.001
7	WBC P-WBC M (x10^9^/l)	.04	1.2	-.24	.32	.759
8	NE P-NE M (%)	-.45	2.94	-1.13	.21	.182
9	LY P-LY M (%)	.20	3.30	-.55	.96	.590
10	MO P-MO M (%)	.30	1.37	-.01	.62	.057
11	EO P-EO M (%)	.28	3.46	-.50	1.08	.472
12	BA P-BA M (%)	.01	.33	-.06	.08	.754
13	PLT P-PLT M (x10^9^/l)	.13	.26	.07	.19	<0.001
14	MPV P-MPV M (fl)	-.18	.64	-.32	-.03	.017

P: pneumatic tube samples; M: hand delivered samples; RBC: red blood cells count; HB: hemoglobin; MCV: mean corpuscular volume; MCH: mean corpuscular hemoglobin; RDW: red cell distribution width; WBC: white blood cell count; NE: neutrophils; LY: lymphocytes; MO: monocytes; EO: eosinophils; BA: basophils; PLT: platelets; MPV: mean platelet volume.

**Table 2:** Summary of the differences in PT and APTT between pneumatic tube samples and hand delivered samples.

S. no.	Paired samples	Mean difference	Standard deviation	95% confidence interval of mean difference		*P* value
				Lower	Upper	
1	PT P-PT M (secs)	-.38	1.3	-.95	.18	.17
2	APTT P-APTT M (secs)	-.33	3.5	-1.7	1.1	.63

P: pneumatic tube samples; M: hand delivered samples; PT: prothrombin time; APTT: activated partial thromboplastin time.

## Data Availability

The data used to support the findings of this study are available from the corresponding author upon request.
